# 2-Amino-7,7-dimethyl-5-oxo-4-(*p*-tol­yl)-5,6,7,8-tetra­hydro-4*H*-chromene-3-carbonitrile

**DOI:** 10.1107/S1600536812033570

**Published:** 2012-07-28

**Authors:** Sumati Anthal, Goutam Brahmachari, Sujay Laskar, Bubun Banerjee, Rajni Kant, Vivek K. Gupta

**Affiliations:** a Post-Graduate Department of Physics & Electronics, University of Jammu, Jammu Tawi 180 006, India; b Laboratory of Natural Products & Organic Synthesis, Department of Chemistry, Visva-Bharati University, Santiniketan 731 235, West Bengal, India

## Abstract

In the title mol­ecule, C_19_H_20_N_2_O_2_, the cyclo­hexene ring adopts a sofa conformation, while the pyran ring adopts a flattened boat conformation. In the crystal, mol­ecules are linked by N—H⋯N and N—H⋯O hydrogen bonds, forming a two-dimensional network parallel to (010).

## Related literature

For background to compounds containing the 4*H*-pyran unit, see: Brahmachari (2010 ▶); Hatakeyama *et al.* (1988 ▶). For the biological activity of compounds containing a tetra­hydro­benzo[*b*]pyran ring system, see: Andreani & Lapi (1960 ▶); Bonsignore *et al.* (1993 ▶); Brahmachari (2011 ▶); Konkoy *et al.* (2001 ▶). For 2-amino-4*H*-pyrans as photoactive materials, see: Armetso *et al.* (1989 ▶). For the synthesis of related compounds, see: Jin *et al.* (2004 ▶); Balalaie *et al.* (2007 ▶). For related structures, see: Tu *et al.* (2001 ▶); Wang (2011 ▶). For ring conformations, see: Duax *et al.* (1975 ▶).
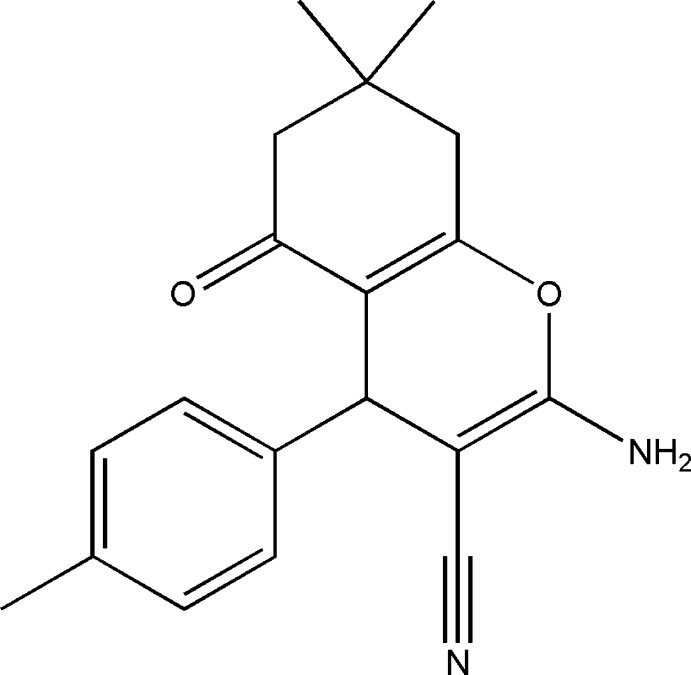


## Experimental

### 

#### Crystal data

C_19_H_20_N_2_O_2_
*M* *_r_* = 308.37
Monoclinic, 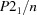

*a* = 9.4622 (3) Å
*b* = 16.8820 (5) Å
*c* = 10.8301 (4) Å
β = 111.842 (4)°
*V* = 1605.82 (9) Å^3^
*Z* = 4
Mo *K*α radiation
μ = 0.08 mm^−1^
*T* = 293 K
0.30 × 0.20 × 0.20 mm

#### Data collection

Oxford Diffraction Xcalibur Sapphire3 diffractometer
Absorption correction: multi-scan (*CrysAlis PRO*; Oxford Diffraction, 2010 ▶) *T*_min_ = 0.862, *T*_max_ = 1.000
18449 measured reflections
3149 independent reflections
2428 reflections with *I* > 2σ(*I*)
*R*_int_ = 0.037

#### Refinement

*R*[*F* ^2^ > 2σ(*F* ^2^)] = 0.046
*wR*(*F* ^2^) = 0.115
*S* = 1.05
3149 reflections
219 parameters
H atoms treated by a mixture of independent and constrained refinement
Δρ_max_ = 0.20 e Å^−3^
Δρ_min_ = −0.22 e Å^−3^

### 

Data collection: *CrysAlis PRO* (Oxford Diffraction, 2010 ▶); cell refinement: *CrysAlis PRO*; data reduction: *CrysAlis PRO*; program(s) used to solve structure: *SHELXS97* (Sheldrick, 2008 ▶); program(s) used to refine structure: *SHELXL97* (Sheldrick, 2008 ▶); molecular graphics: *ORTEP-3* (Farrugia, 1997 ▶); software used to prepare material for publication: *PLATON* (Spek, 2009 ▶).

## Supplementary Material

Crystal structure: contains datablock(s) I, global. DOI: 10.1107/S1600536812033570/lh5503sup1.cif

Structure factors: contains datablock(s) I. DOI: 10.1107/S1600536812033570/lh5503Isup2.hkl

Supplementary material file. DOI: 10.1107/S1600536812033570/lh5503Isup3.cml

Additional supplementary materials:  crystallographic information; 3D view; checkCIF report

## Figures and Tables

**Table 1:** Hydrogen-bond geometry (Å, °)

*D*—H⋯*A*	*D*—H	H⋯*A*	*D*⋯*A*	*D*—H⋯*A*
N13—H131⋯O5^i^	0.89 (2)	2.06 (2)	2.913 (2)	161 (2)
N13—H132⋯N15^ii^	0.87 (2)	2.35 (2)	3.168 (2)	156 (2)

Symmetry codes: (i) 
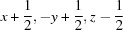
; (ii) 
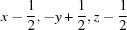
.

**Fig. 1.**
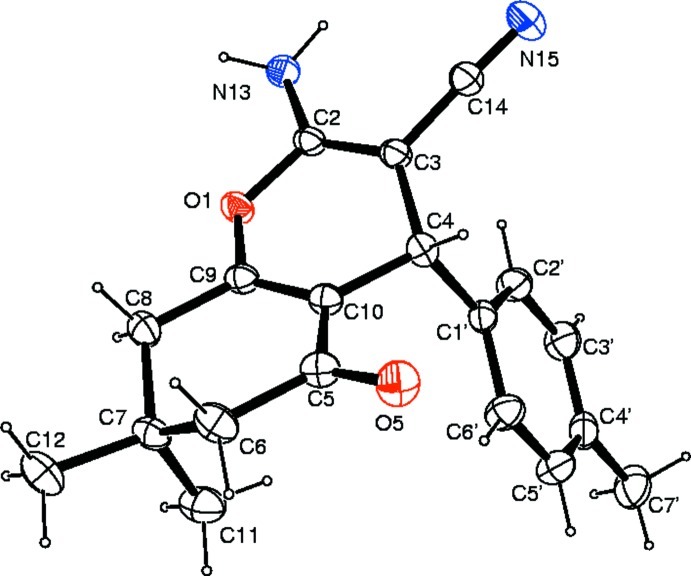
The molecular structure of the title compound with ellipsoids are drawn at the 40% probability level. H atoms are shown as small spheres of arbitrary radii.

**Fig. 2.**
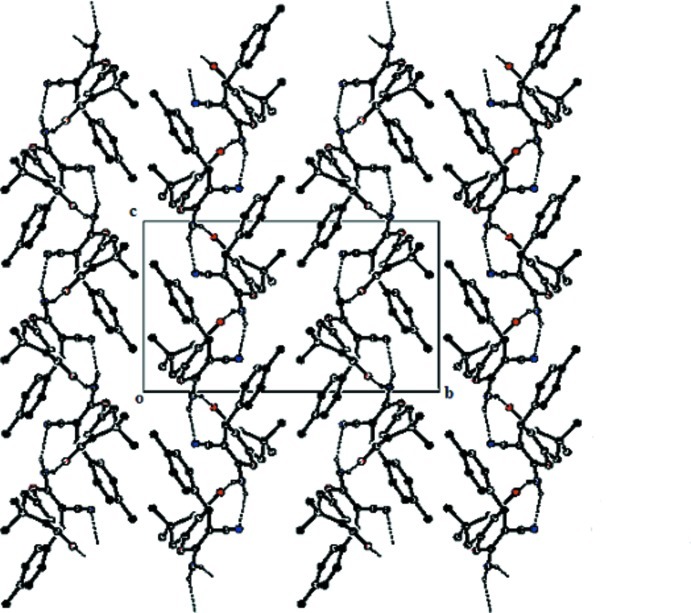
The packing arrangement of molecules viewed along the a axis. Hydrogen bonds are shown as dashed lines.

## supplementary crystallographic information

### Comment

4*H*-Pyran units constitute structural features of a broad range of bioactive natural products (Brahmachari, 2010; Hatakeyama *et al.*, 1988). The tetrahydrobenzo[*b*]pyran ring is of particular interest because compounds bearing this structural motif exhibit diverse biological activities such as spasmolytic, anticancer and anti-anaphylactin agents (Andreani *et al.*, 1960; Bonsignore *et al.*, 1993), anti-Alzheimer's disease (Brahmachari, 2011), anti-Huntington's disease, anti-Parkinson's disease and anti-HIV (Konkoy *et al.*, 2001). 2-Amino-4*H*-pyrans have also been found to be useful as photoactive materials (Armetso *et al.*, 1989). Hence, investigation of the structural features of biologically relevant tetrahydrobenzo[*b*]pyran derivatives is of both scientific and practical interest. In continuation of our efforts to develop useful synthetic protocols for biologically significant molecules, we herein report an efficient and environmentally benign synthesis and the crystal structure of the title compound. The bond lengths and angles of the title compound are normal and correspond to those observed in related structures (Tu *et al.*, 2001; Wang, 2011). The cyclohexene ring adopts a sofa conformation while the pyran ring adopts a flattened boat conformation with asymmetry parameters [ΔCs(C7) = 5.71] and [ΔCs (O1—C4) = 0.08; ΔCs (C2—C3) = 9.8)] respectively (Duax *et al.*, 1975). In the crystal structure, intermolecular N—H···N and N—H···O hydrogen bonds link the molecules into a two-dimensional network parallel to (010) (Fig.2).

### Experimental

The synthesis of the title compounds was carried out *via* one-pot multi-component reaction in aqueous ethanol using low-cost and environmentally benign sodium formate as catalyst at room temperature. An oven-dried screw cap test tube was charged with a magnetic stir bar, *p*-methylbenzaldehyde (0.12 g, 1 mmol), malononitrile (0.066 g, 1 mmol) and sodium formate (0.136 g, 20 mol %) in 5 ml aqueous ethanol. The reaction mixture was then started to stir vigorously and after 20 min 1 mmol of dimedone (0.14 g) was added, and continued to stir. After completion of the overall reaction (2 h) as monitored by TLC, a white solid was precipitated out, filtered off, and washed with aqueous ethanol. Recrystallization from ethanol afforded the title compound as white block-shaped crystals (252 mg, yield 82%) with the m.p. 492–494 K (lit. 492–495 K) (Balalaie *et al.*, 2007). Rf 0.82 (EtOAc). White solid; FT—IR (KBr) νmax 3375, 3256, 3180, 2961, 2920, 2885, 2187, 1677, 1637, 1607, 1512, 1460, 1414, 1366, 1215, 1137, 1030, 825, 764, 565 cm-1; 1*H*-NMR (DMSO-d6, 400 MHz) & 13 C-NMR (DMSO-d6; 100 MHz) data are in excellent agreement with literature values (Jin *et al.*, 2004; Balalaie *et al.*, 2007); TOF-MS: calculated for C_19_H_20_N_2_O_2_Na 331.1422 [*M* + Na]+; found 331.1426. For crystallization 60 mg of the compound was dissolved in 20 ml mixture of ethanol and water (5:1) and left for several days at ambient temperature which yielded white block-shaped crystals.

### Refinement

H131 and H132 attached to N13 were located in a difference map and refined isotropically. The remaining H atoms were positioned geometrically and were treated as riding on their parent C atoms, with C—H distances of 0.93–0.98 Å; and with *U*_iso_(H) = 1.2*U*_eq_(C), except for the methyl groups where *U*_iso_(H) = 1.5*U*_eq_(C).

### Crystal data

**Table:**

C_19_H_20_N_2_O_2_	*F*(000) = 656
*M**_r_* = 308.37	*D*_x_ = 1.276 Mg m^−^^3^
Monoclinic, *P*2_1_/*n*	Mo *K*α radiation, λ = 0.71073 Å
Hall symbol: -P 2yn	Cell parameters from 7806 reflections
*a* = 9.4622 (3) Å	θ = 3.6–29.1°
*b* = 16.8820 (5) Å	µ = 0.08 mm^−^^1^
*c* = 10.8301 (4) Å	*T* = 293 K
β = 111.842 (4)°	Block, white
*V* = 1605.82 (9) Å^3^	0.30 × 0.20 × 0.20 mm
*Z* = 4	

### Data collection

**Table:**

Oxford Diffraction Xcalibur Sapphire3 diffractometer	3149 independent reflections
Radiation source: fine-focus sealed tube	2428 reflections with *I* > 2σ(*I*)
Graphite monochromator	*R*_int_ = 0.037
Detector resolution: 16.1049 pixels mm^-1^	θ_max_ = 26.0°, θ_min_ = 3.6°
ω scans	*h* = −11→11
Absorption correction: multi-scan (*CrysAlis PRO*; Oxford Diffraction, 2010)	*k* = −20→20
*T*_min_ = 0.862, *T*_max_ = 1.000	*l* = −13→13
18449 measured reflections	

### Refinement

**Table:**

Refinement on *F*^2^	Primary atom site location: structure-invariant direct methods
Least-squares matrix: full	Secondary atom site location: difference Fourier map
*R*[*F*^2^ > 2σ(*F*^2^)] = 0.046	Hydrogen site location: inferred from neighbouring sites
*wR*(*F*^2^) = 0.115	H atoms treated by a mixture of independent and constrained refinement
*S* = 1.05	*w* = 1/[σ^2^(*F*_o_^2^) + (0.0501*P*)^2^ + 0.4013*P*] where *P* = (*F*_o_^2^ + 2*F*_c_^2^)/3
3149 reflections	(Δ/σ)_max_ = 0.001
219 parameters	Δρ_max_ = 0.20 e Å^−^^3^
0 restraints	Δρ_min_ = −0.22 e Å^−^^3^

### Special details

**Table:**

Experimental. Absorption correction: *CrysAlis PRO*, Oxford Diffraction Ltd., Version 1.171.34.40 (release 27–08-2010 CrysAlis171. NET) (compiled Aug 27 2010,11:50:40) Empirical absorption correction using spherical harmonics, implemented in SCALE3 ABSPACK scaling algorithm.
Geometry. All e.s.d.'s (except the e.s.d. in the dihedral angle between two l.s. planes) are estimated using the full covariance matrix. The cell e.s.d.'s are taken into account individually in the estimation of e.s.d.'s in distances, angles and torsion angles; correlations between e.s.d.'s in cell parameters are only used when they are defined by crystal symmetry. An approximate (isotropic) treatment of cell e.s.d.'s is used for estimating e.s.d.'s involving l.s. planes.
Refinement. Refinement of *F*^2^ against ALL reflections. The weighted *R*-factor *wR* and goodness of fit *S* are based on *F*^2^, conventional *R*-factors *R* are based on *F*, with *F* set to zero for negative *F*^2^. The threshold expression of *F*^2^ > σ(*F*^2^) is used only for calculating *R*-factors(gt) *etc*. and is not relevant to the choice of reflections for refinement. *R*-factors based on *F*^2^ are statistically about twice as large as those based on *F*, and *R*- factors based on ALL data will be even larger.

### Fractional atomic coordinates and isotropic or equivalent isotropic displacement parameters (Å^2^)

**Table:**

	*x*	*y*	*z*	*U*_iso_*/*U*_eq_	
O1	0.62616 (12)	0.12747 (6)	0.06792 (11)	0.0370 (3)	
C1'	0.87094 (17)	0.17332 (9)	0.42111 (15)	0.0324 (4)	
C2'	1.00784 (18)	0.14575 (11)	0.41998 (17)	0.0433 (4)	
H2'	1.0343	0.1568	0.3472	0.052*	
C3'	1.1067 (2)	0.10204 (11)	0.52474 (19)	0.0498 (5)	
H3'	1.1978	0.0842	0.5206	0.060*	
C4'	1.0732 (2)	0.08433 (10)	0.63476 (17)	0.0442 (4)	
C5'	0.9371 (2)	0.11238 (11)	0.63673 (18)	0.0504 (5)	
H5'	0.9118	0.1018	0.7103	0.060*	
C6'	0.8370 (2)	0.15591 (11)	0.53232 (17)	0.0456 (4)	
H6'	0.7459	0.1737	0.5367	0.055*	
C7'	1.1810 (2)	0.03593 (13)	0.7475 (2)	0.0645 (6)	
H7'1	1.1854	−0.0172	0.7175	0.097*	
H7'2	1.1456	0.0350	0.8199	0.097*	
H7'3	1.2807	0.0592	0.7770	0.097*	
C2	0.74563 (17)	0.17699 (9)	0.07890 (15)	0.0324 (4)	
C3	0.80680 (17)	0.22511 (9)	0.18489 (15)	0.0325 (4)	
C4	0.75981 (17)	0.22126 (9)	0.30428 (15)	0.0325 (4)	
H4	0.7564	0.2755	0.3353	0.039*	
O5	0.53082 (14)	0.26138 (8)	0.40897 (12)	0.0488 (3)	
C5	0.49500 (18)	0.21056 (10)	0.32139 (16)	0.0372 (4)	
C6	0.33944 (19)	0.17324 (11)	0.27258 (19)	0.0455 (4)	
H6A	0.2688	0.2083	0.2076	0.055*	
H6B	0.3062	0.1686	0.3469	0.055*	
C7	0.33113 (18)	0.09159 (10)	0.20960 (17)	0.0386 (4)	
C8	0.39789 (18)	0.09955 (11)	0.10153 (17)	0.0406 (4)	
H8A	0.4110	0.0472	0.0705	0.049*	
H8B	0.3267	0.1284	0.0267	0.049*	
C9	0.54697 (16)	0.14144 (9)	0.14978 (15)	0.0321 (4)	
C10	0.60023 (17)	0.18777 (9)	0.25695 (15)	0.0312 (4)	
C11	0.4198 (2)	0.03164 (12)	0.3151 (2)	0.0568 (5)	
H11A	0.5238	0.0486	0.3562	0.085*	
H11B	0.4164	−0.0192	0.2743	0.085*	
H11C	0.3752	0.0277	0.3813	0.085*	
C12	0.1651 (2)	0.06452 (13)	0.1450 (2)	0.0579 (5)	
H12A	0.1612	0.0141	0.1030	0.087*	
H12B	0.1084	0.1027	0.0797	0.087*	
H12C	0.1218	0.0598	0.2120	0.087*	
N13	0.78732 (18)	0.16693 (10)	−0.02573 (15)	0.0432 (4)	
C14	0.92224 (19)	0.27927 (10)	0.18699 (16)	0.0396 (4)	
N15	1.01708 (19)	0.32308 (10)	0.19332 (17)	0.0578 (5)	
H131	0.864 (2)	0.1956 (12)	−0.0300 (19)	0.055 (6)*	
H132	0.716 (2)	0.1541 (12)	−0.101 (2)	0.059 (6)*	

### Atomic displacement parameters (Å^2^)

**Table:**

	*U*^11^	*U*^22^	*U*^33^	*U*^12^	*U*^13^	*U*^23^
O1	0.0355 (6)	0.0427 (6)	0.0391 (6)	−0.0084 (5)	0.0212 (5)	−0.0064 (5)
C1'	0.0317 (8)	0.0332 (8)	0.0311 (8)	−0.0050 (6)	0.0102 (6)	−0.0051 (6)
C2'	0.0344 (9)	0.0567 (11)	0.0404 (9)	0.0021 (8)	0.0159 (7)	0.0041 (8)
C3'	0.0340 (9)	0.0609 (12)	0.0502 (11)	0.0054 (8)	0.0108 (8)	0.0035 (9)
C4'	0.0430 (10)	0.0388 (10)	0.0396 (10)	−0.0060 (8)	0.0024 (8)	−0.0022 (7)
C5'	0.0597 (12)	0.0567 (12)	0.0361 (9)	−0.0019 (9)	0.0194 (9)	0.0032 (8)
C6'	0.0459 (10)	0.0552 (11)	0.0400 (10)	0.0052 (8)	0.0210 (8)	0.0014 (8)
C7'	0.0638 (13)	0.0584 (13)	0.0519 (12)	0.0001 (10)	−0.0010 (10)	0.0096 (10)
C2	0.0269 (7)	0.0373 (9)	0.0351 (8)	−0.0002 (6)	0.0139 (6)	0.0059 (7)
C3	0.0289 (8)	0.0349 (9)	0.0345 (8)	−0.0019 (6)	0.0127 (6)	0.0044 (6)
C4	0.0311 (8)	0.0311 (8)	0.0366 (8)	−0.0005 (6)	0.0141 (7)	−0.0034 (6)
O5	0.0463 (7)	0.0556 (8)	0.0513 (7)	0.0043 (6)	0.0260 (6)	−0.0126 (6)
C5	0.0367 (9)	0.0374 (9)	0.0412 (9)	0.0067 (7)	0.0188 (7)	0.0033 (7)
C6	0.0359 (9)	0.0487 (11)	0.0608 (11)	0.0036 (8)	0.0284 (8)	−0.0003 (8)
C7	0.0317 (8)	0.0417 (10)	0.0482 (10)	−0.0005 (7)	0.0215 (7)	0.0046 (7)
C8	0.0322 (8)	0.0479 (10)	0.0422 (9)	−0.0069 (7)	0.0146 (7)	−0.0028 (7)
C9	0.0289 (8)	0.0362 (9)	0.0343 (8)	0.0034 (6)	0.0156 (7)	0.0046 (7)
C10	0.0290 (8)	0.0327 (8)	0.0345 (8)	0.0027 (6)	0.0147 (6)	0.0018 (6)
C11	0.0588 (12)	0.0526 (12)	0.0643 (13)	0.0024 (9)	0.0290 (10)	0.0162 (10)
C12	0.0373 (10)	0.0653 (13)	0.0772 (14)	−0.0096 (9)	0.0283 (10)	−0.0017 (11)
N13	0.0352 (8)	0.0630 (10)	0.0359 (8)	−0.0091 (7)	0.0184 (7)	0.0004 (7)
C14	0.0355 (9)	0.0425 (10)	0.0399 (9)	−0.0034 (8)	0.0132 (7)	0.0035 (7)
N15	0.0501 (9)	0.0570 (10)	0.0648 (11)	−0.0187 (8)	0.0198 (8)	0.0028 (8)

### Geometric parameters (Å, º)

**Table:**

O1—C2	1.3751 (18)	O5—C5	1.229 (2)
O1—C9	1.3774 (18)	C5—C10	1.464 (2)
C1'—C2'	1.381 (2)	C5—C6	1.505 (2)
C1'—C6'	1.389 (2)	C6—C7	1.527 (2)
C1'—C4	1.540 (2)	C6—H6A	0.9700
C2'—C3'	1.384 (2)	C6—H6B	0.9700
C2'—H2'	0.9300	C7—C11	1.523 (2)
C3'—C4'	1.375 (3)	C7—C8	1.529 (2)
C3'—H3'	0.9300	C7—C12	1.532 (2)
C4'—C5'	1.380 (3)	C8—C9	1.488 (2)
C4'—C7'	1.508 (2)	C8—H8A	0.9700
C5'—C6'	1.385 (3)	C8—H8B	0.9700
C5'—H5'	0.9300	C9—C10	1.333 (2)
C6'—H6'	0.9300	C11—H11A	0.9600
C7'—H7'1	0.9600	C11—H11B	0.9600
C7'—H7'2	0.9600	C11—H11C	0.9600
C7'—H7'3	0.9600	C12—H12A	0.9600
C2—N13	1.342 (2)	C12—H12B	0.9600
C2—C3	1.348 (2)	C12—H12C	0.9600
C3—C14	1.418 (2)	N13—H131	0.88 (2)
C3—C4	1.517 (2)	N13—H132	0.87 (2)
C4—C10	1.512 (2)	C14—N15	1.145 (2)
C4—H4	0.9800		
			
C2—O1—C9	117.83 (12)	C5—C6—C7	114.58 (13)
C2'—C1'—C6'	117.19 (15)	C5—C6—H6A	108.6
C2'—C1'—C4	121.74 (14)	C7—C6—H6A	108.6
C6'—C1'—C4	121.08 (14)	C5—C6—H6B	108.6
C1'—C2'—C3'	121.49 (17)	C7—C6—H6B	108.6
C1'—C2'—H2'	119.3	H6A—C6—H6B	107.6
C3'—C2'—H2'	119.3	C11—C7—C6	110.02 (15)
C4'—C3'—C2'	121.53 (17)	C11—C7—C8	111.30 (14)
C4'—C3'—H3'	119.2	C6—C7—C8	107.18 (14)
C2'—C3'—H3'	119.2	C11—C7—C12	109.22 (15)
C3'—C4'—C5'	117.14 (16)	C6—C7—C12	110.29 (14)
C3'—C4'—C7'	120.99 (18)	C8—C7—C12	108.80 (15)
C5'—C4'—C7'	121.87 (18)	C9—C8—C7	112.37 (14)
C4'—C5'—C6'	121.89 (17)	C9—C8—H8A	109.1
C4'—C5'—H5'	119.1	C7—C8—H8A	109.1
C6'—C5'—H5'	119.1	C9—C8—H8B	109.1
C5'—C6'—C1'	120.77 (17)	C7—C8—H8B	109.1
C5'—C6'—H6'	119.6	H8A—C8—H8B	107.9
C1'—C6'—H6'	119.6	C10—C9—O1	122.74 (14)
C4'—C7'—H7'1	109.5	C10—C9—C8	125.90 (14)
C4'—C7'—H7'2	109.5	O1—C9—C8	111.36 (13)
H7'1—C7'—H7'2	109.5	C9—C10—C5	117.73 (14)
C4'—C7'—H7'3	109.5	C9—C10—C4	121.39 (14)
H7'1—C7'—H7'3	109.5	C5—C10—C4	120.70 (14)
H7'2—C7'—H7'3	109.5	C7—C11—H11A	109.5
N13—C2—C3	128.83 (15)	C7—C11—H11B	109.5
N13—C2—O1	109.91 (14)	H11A—C11—H11B	109.5
C3—C2—O1	121.24 (14)	C7—C11—H11C	109.5
C2—C3—C14	119.09 (15)	H11A—C11—H11C	109.5
C2—C3—C4	122.04 (13)	H11B—C11—H11C	109.5
C14—C3—C4	118.83 (14)	C7—C12—H12A	109.5
C10—C4—C3	107.42 (12)	C7—C12—H12B	109.5
C10—C4—C1'	111.87 (12)	H12A—C12—H12B	109.5
C3—C4—C1'	113.31 (13)	C7—C12—H12C	109.5
C10—C4—H4	108.0	H12A—C12—H12C	109.5
C3—C4—H4	108.0	H12B—C12—H12C	109.5
C1'—C4—H4	108.0	C2—N13—H131	118.5 (13)
O5—C5—C10	120.66 (15)	C2—N13—H132	116.8 (14)
O5—C5—C6	121.07 (15)	H131—N13—H132	116.9 (19)
C10—C5—C6	118.19 (15)	N15—C14—C3	177.67 (18)
			
C6'—C1'—C2'—C3'	0.5 (3)	C10—C5—C6—C7	26.8 (2)
C4—C1'—C2'—C3'	−179.38 (16)	C5—C6—C7—C11	67.88 (19)
C1'—C2'—C3'—C4'	−0.2 (3)	C5—C6—C7—C8	−53.27 (19)
C2'—C3'—C4'—C5'	−0.4 (3)	C5—C6—C7—C12	−171.57 (15)
C2'—C3'—C4'—C7'	179.35 (17)	C11—C7—C8—C9	−71.62 (19)
C3'—C4'—C5'—C6'	0.6 (3)	C6—C7—C8—C9	48.72 (18)
C7'—C4'—C5'—C6'	−179.09 (18)	C12—C7—C8—C9	167.98 (15)
C4'—C5'—C6'—C1'	−0.3 (3)	C2—O1—C9—C10	−15.5 (2)
C2'—C1'—C6'—C5'	−0.2 (3)	C2—O1—C9—C8	163.73 (13)
C4—C1'—C6'—C5'	179.64 (15)	C7—C8—C9—C10	−19.0 (2)
C9—O1—C2—N13	−166.15 (13)	C7—C8—C9—O1	161.72 (13)
C9—O1—C2—C3	15.4 (2)	O1—C9—C10—C5	168.43 (13)
N13—C2—C3—C14	6.5 (3)	C8—C9—C10—C5	−10.7 (2)
O1—C2—C3—C14	−175.37 (14)	O1—C9—C10—C4	−6.7 (2)
N13—C2—C3—C4	−171.42 (16)	C8—C9—C10—C4	174.14 (15)
O1—C2—C3—C4	6.7 (2)	O5—C5—C10—C9	−170.15 (15)
C2—C3—C4—C10	−25.3 (2)	C6—C5—C10—C9	6.7 (2)
C14—C3—C4—C10	156.78 (14)	O5—C5—C10—C4	5.0 (2)
C2—C3—C4—C1'	98.75 (17)	C6—C5—C10—C4	−178.09 (14)
C14—C3—C4—C1'	−79.14 (18)	C3—C4—C10—C9	25.20 (19)
C2'—C1'—C4—C10	129.01 (16)	C1'—C4—C10—C9	−99.76 (17)
C6'—C1'—C4—C10	−50.87 (19)	C3—C4—C10—C5	−149.80 (14)
C2'—C1'—C4—C3	7.4 (2)	C1'—C4—C10—C5	85.24 (17)
C6'—C1'—C4—C3	−172.49 (15)	C2—C3—C14—N15	−157 (5)
O5—C5—C6—C7	−156.36 (16)	C4—C3—C14—N15	20 (5)

### Hydrogen-bond geometry (Å, º)

**Table:**

*D*—H···*A*	*D*—H	H···*A*	*D*···*A*	*D*—H···*A*
N13—H131···O5^i^	0.89 (2)	2.06 (2)	2.913 (2)	161 (2)
N13—H132···N15^ii^	0.87 (2)	2.35 (2)	3.168 (2)	156 (2)

Symmetry codes: (i) *x*+1/2, −*y*+1/2, *z*−1/2; (ii) *x*−1/2, −*y*+1/2, *z*−1/2.
